# Route of Infection Influences Zika Virus Shedding in a Guinea Pig Model

**DOI:** 10.3390/cells8111437

**Published:** 2019-11-14

**Authors:** Ashley E. Saver, Stephanie A. Crawford, Jonathan D. Joyce, Andrea S. Bertke

**Affiliations:** 1 Virginia-Maryland College of Veterinary Medicine, Virginia Polytechnic Institute & State University, Blacksburg, VA 24061, USA; aes27@vt.edu (A.E.S.); stepc08@vt.edu (S.A.C.); 2 Department of Population Health Sciences, Virginia-Maryland College of Veterinary Medicine, Virginia Polytechnic Institute & State University, Blacksburg, VA 24061, USA; jjoyce84@vt.edu

**Keywords:** Zika virus, ZIKV, virus host interactions, pathogenesis, MR766, guinea pig, subcutaneous, vaginal, sexual transmission, virus transmission

## Abstract

Due to the recent epidemic of Zika virus (ZIKV) infection and resulting sequelae, as well as concerns about both the sexual and vertical transmission of the virus, renewed attention has been paid to the pathogenesis of this unique arbovirus. Numerous small animal models have been used in various ZIKV pathogenicity studies, however, they are often performed using immunodeficient or immunosuppressed animals, which may impact disease progression in a manner not relevant to immunocompetent humans. The use of immunocompetent animal models, such as macaques, is constrained by small sample sizes and the need for specialized equipment/staff. Here we report the establishment of ZIKV infection in an immunocompetent small animal model, the guinea pig, using both subcutaneous and vaginal routes of infection to mimic mosquito-borne and sexual transmission. Guinea pigs developed clinical signs consistent with mostly asymptomatic and mild disease observed in humans. We demonstrate that the route of infection does not significantly alter viral tissue tropism but does impact mucosal shedding mechanics. We also demonstrate persistent infection in sensory and autonomic ganglia, identifying a previously unrecognized niche of viral persistence that could contribute to viral shedding in secretions. We conclude that the guinea pig represents a useful and relevant model for ZIKV pathogenesis.

## 1. Introduction

Zika virus (ZIKV), discovered in the Ugandan Zika forest in 1947, is a single-stranded RNA arbovirus of the genus *Flavivirus* and the family Flaviviridae [1,2]. Prior to 2007, only 14 human cases of ZIKV infection had been reported. However, in 2007, the first major epidemic of ZIKV, with 185 confirmed cases, occurred in the Yap Islands of the Federated States of Micronesia [3,4]. Since then, ZIKV has spread to 30+ countries, with millions of suspected cases, and has gained international attention due to an association with microcephaly and Guillain-Barré Syndrome (GBS) [5,6,7,8,9]. Subsequently, ZIKV has been identified as a significant global health threat.

ZIKV is primarily transmitted by mosquitoes. However, it can also be transmitted sexually or by blood transfusion [10,11,12]. After inoculation from an infected mosquito, the virus replicates in tissues local to the bite, drains to local lymph nodes, and then spreads hematogenously to secondary replication sites [13]. In adults, most infections (~80%) are asymptomatic, with only about 20% of infections developing a self-limiting illness. Symptoms vary in severity, and may include fever, headache, maculopapular rash, arthralgia, myalgia, fatigue, and conjunctivitis [14]. Additionally, ZIKV infection during pregnancy can cross the placenta, where it targets neural stem and progenitor cells in the developing fetus, leading to microcephaly, lissencephaly, and cognitive deficits, as well as ocular impairments such as chorioretinal atrophy and optic nerve disorders [15,16,17].

ZIKV is the only arbovirus known to be transmitted sexually [18]. Sexual transmission has been reported from male to female, male to male, and female to male, indicating that infectious virus persists in both semen (up to four months [18]) and vaginal secretions (up to six months [19]) [18,20,21,22,23,24,25,26,27,28]. However, the site of ZIKV persistence, leading to viral shedding in the genital secretions of males and females, is not clear. Although ZIKV has been reported to persist in testes, evidence of viral shedding in semen of vasectomized males suggests an additional site of persistence [29,30,31]. In women, the site of persistence has not been determined. We recently showed that ZIKV persistently infects primary adult cultured sensory neurons of the lumbosacral dorsal root ganglia (LS-DRG), which innervate the genitourinary tract (GUT), suggesting a potential alternative reservoir for viral shedding in urine and genital secretions [32]. The pathogenesis of ZIKV after sexual transmission has not been studied extensively, but sexual transmission may result in different routes of spread within the host and potentially alter tissue tropism when compared to mosquito-borne transmission.

Efforts to understand the pathogenesis of ZIKV following mosquito-borne and sexual transmission have led to the development of various animal models. Several studies have shown that immunocompetent adult wild-type mice have minimal susceptibility to ZIKV infection and demonstrate different disease manifestations than humans [33]. Thus, more recent studies have primarily used immunocompromised animals, such as mice lacking interferon (IFN) or IFN receptors, or immunocompetent mice treated with IFN-blocking antibodies [34,35,36,37,38,39,40]. Neonatal wild-type mice are susceptible to ZIKV infection, but they are also immunocompromised since rodents do not develop a mature immune response until at least one month of age [36,41,42,43]. Non-human primate models have provided valuable information [44,45,46,47,48]. However, non-human primate studies are limited in statistical power since relatively few animals can be used in studies. Additionally, non-human primate studies are expensive to perform and are limited to facilities that have the necessary infrastructure to house these animals. More recently, several studies have explored the use of swine as a model of ZIKV infection; however, most infected swine do not exhibit clinical signs and have demonstrated only low levels of viremia [49,50]. Additionally, swine pose similar constraints as non-human primates, as they require more space and are more expensive than small animal models. Thus, an immunocompetent small animal model is needed to study ZIKV pathogenesis by different routes of infection.

Guinea pigs (*Cavia porcellus*) have served as reliable models of flavivirus infection, and due to their physiologic similarities to human immune responses and symptoms, are also used as a genital infection model for several viruses [51,52,53]. Furthermore, guinea pigs are used as a model for cytomegalovirus (CMV) congenital syndrome, which causes similar fetal anomalies as ZIKV [54,55]. To date, five studies have reported the outcome of ZIKV infection in guinea pigs, with varying results. Subcutaneous (SQ) inoculation of strain PRVABC59 (Puerto Rico) resulted in fever, lethargy, hunching, ruffled fur, and decreased mobility, correlating with viremia and viral replication in spleen and brain [56]. Similarly, SQ inoculation of male guinea pigs with strain GZ01 (Venezuela) or FSS13025 (Cambodia) resulted in viremia and robust viral secretion in saliva and tears, as well as transmission to naïve co-caged mates, but no overt signs of disease [57]. However, intracerebral infection with strain MR766 (Uganda) or intraperitoneal inoculation with strain ArD41525 (Senegal) or CPC-0740 (Philippines) failed to produce signs of infection or viremia [1,58]. An additional study assessed fetal impact of mid-gestation infection in guinea pigs, finding viremia and robust antibody response following SQ inoculation with strain H/PF/2013 (French Polynesia) without effects on pups [59]. Although these studies produced variable results, likely due to differences in ZIKV strain, inoculum size, and inoculation route, none of these studies assessed vaginal infection or directly compared different routes of infection. Therefore, we evaluated the use of guinea pigs as an immunocompetent small animal model of ZIKV infection by both subcutaneous (SQ) and vaginal (VAG) inoculation routes, simulating mosquito-borne and sexual transmission, to compare pathogenesis, tissues sites of viral persistence and viral shedding in bodily secretions following different routes of infection.

## 2. Materials and Methods

### 2.1. Ethics Statement

This study was carried out according to the Animal Welfare Act (US Department of Agriculture, Washington DC, USA) and the Public Health Service (PHS) Policy on Humane Care and Use of Laboratory Animals after approval by the Institutional Animal Care and Use Committee (IACUC) of Virginia Polytechnic Institute and State University (Protocol Number: 17-124 approved 7/21/2017).

### 2.2. Virus

African lineage ZIKV MR766 (GenBank accession number: AY632553), recovered from a sentinel rhesus macaque in Uganda in 1947, was used for inoculations (BEI Resources, Manassas, VA, USA). Viral stocks were produced by passage once in Vero E6 cells (CRL-1586, ATCC, Manassas, VA, USA) [60,61]. Viral titer was determined by standard plaque assay on Vero E6 cells [60,61].

### 2.3. Guinea Pig Subcutaneous and Vaginal Infection

After a three day acclimation period, three-week-old female Hartley guinea pigs (Charles River Laboratories, Wilmington, MA, USA) were inoculated under anesthesia with 1 × 10^6^ plaque forming units (PFU) ZIKV MR766 diluted to a total volume of 50 uL in PBS, subcutaneously (SQ, *n* = 6) by injection in the nape of the neck using a tuberculin syringe, or intravaginally (VAG, *n* = 6) by pipette.

Guinea pigs were monitored daily for 24 days for temperature (tympanic), weight, and development of clinical signs of ZIKV infection (fever, lethargy, hunching, ruffled fur, and decreased mobility), using a numerical value (0–5) based on severity of clinical signs, with 0 representing normal parameters and 5 representing severe clinical signs. Vaginal, salivary, and ocular samples were collected daily for 21 days using pre-moistened FLOQSwabs (Copan Diagnostics, Murrieta, CA, USA). Swabs were maintained on ice for the duration of swabbing all animals each day, and then stored at −80 °C until analysis by plaque assay.

Seven days post infection (dpi), two guinea pigs from each group were euthanized to assess acute infection characteristics. Blood samples were collected and centrifuged to separate serum, which was stored at −80 °C until analysis via RT-qPCR. Necropsy was performed immediately following euthanasia and tissues were collected, including spleen, lymph nodes, genitourinary tract (GUT), ovaries, lumbosacral DRG (LS-DRG, innervates the GUT), cervical DRG (C-DRG, innervates the neck), trigeminal ganglia (TG, innervates the face), superior cervical ganglion (SCG, innervates head and neck), ciliary ganglion (CG, motor and sensory innervation to the eye), brain, and eyes. Half of the tissues were fixed in 4% paraformaldehyde (PFA), embedded in optimum cutting temperature (OCT) media, and sectioned for immunofluorescence. The other half of the tissues were frozen for RNA isolation for RT-qPCR. At 37 dpi, allowing five weeks for viral clearance to ensure we had reached convalescence, the remaining guinea pigs were euthanized, necropsied, and samples were collected as described above to assess convalescent characteristics and viral persistence.

### 2.4. ZIKV Shedding

Daily ocular, vaginal and oral swabs were collected from 1–21 dpi, using pre-moistened FLOQswabs. Viral shedding was assessed by standard plaque assay on Vero E6 cells using ocular and vaginal swab samples collected daily 1–21 dpi. Oral swabs were unable to be assessed by plaque assay due to excessive outgrowth of oral microflora.

### 2.5. ZIKV RNA Isolation and qPCR of Clinical Samples

Tissues were homogenized on ice with a Bel-Art hand held tissue homogenizer with sterile pestles (Cole-Parmer, Vernon Hills IL, USA). RNA was extracted using an RNEasy Kit (Qiagen, Germantown, MD, USA) following manufacturer’s protocol. The iScript cDNA Synthesis Kit was used to reverse transcribe RNA into complementary DNA (cDNA) (Bio-Rad, Hercules, CA, USA). Briefly, 20 µL reactions were used for cDNA synthesis (4 µL 5× iScript reaction mix, 1 µL iScript reverse transcriptase, 10 µL nuclease-free water, and 5 µL RNA template). Thermocycler conditions were: 5 min 25 °C; reverse transcription for 30 min 42 °C; 5 min 85 °C. qPCR was conducted on a Viia 7 real-time PCR system (Applied Biosystems, v1.2.4, Foster City, CA, USA) using iTaq Universal SYBR Green Supermix (BioRad) and the previously synthesized cDNA. Ten μL reactions were used (5 μL iTaq supermix, 0.5 μL forward (5′ AAR TAC ACA TAC CAR AAC AAA GTG GT) /reverse (5′ TCC RCT CCC YCT YTG GTC TTG) primer mix (10 μM each), 0.5 μL rRNA primer mix, 1 μL nuclease-free water, 3 μL cDNA). Thermocycler conditions were on fast setting, 20 s 95 °C; 40 cycles of 1 sec 95 °C, 20 sec 60 °C; followed by melt-curve analysis. Resulting Ct values were used to determine number of ZIKV RNA copies/500 ng total RNA based on standards of known quantity.

### 2.6. Immunofluorescence of Clinical Samples

Tissues were fixed in 4% PFA, embedded in OCT media (ThermoFisher, Waltham, MA, USA), and stored at −80 °C until sectioned into 10 µm cryosections using a Leica CM3050-S cryostat (Leica Biosystems, Buffalo Grove, IL, USA), and sections were stored at −80 °C until immunostaining. Sections were immunostained for ZIKV using a mouse-anti-ZIKV Envelope (E) protein primary antibody (FL0006, Kerafast, Boston, MA, USA), and visualized with a donkey-anti-mouse-AlexaFluor 488 secondary antibody (ab150101, Abcam, Cambridge, MA, USA). Sensory neurons were immunostained with anti-PGP9.5 (NB300-675, Novus Biologicals, Centennial, CO, USA) and satellite glial cells with anti-glutamine synthetase (ab73593, Abcam, Cambridge MA, USA) antibodies, followed by species-specific secondary antibodies conjugated to AlexaFluor 488 (Fisher Scientific, Hamptom, NH, USA). CNS neurons were immunostained with anti-NeuN conjugated to AlexaFluor 488 (ABN78A4, EMD Millipore, Burlington, MA, USA). Slides were treated with SlowFade Gold Antifade Mountant (ThermoFisher, Waltham, MA, USA) before cover slipping. Slides were visualized and imaged using an IX71 inverted fluorescence microscope (Olympus Life Sciences, Waltham, MA, USA).

### 2.7. Statistical Analysis

Statistical analyses were performed in Excel (Microsoft Inc., Redmond, WA, USA) using t-tests to compare the two groups. *p*-values are summarized in figures as * < 0.01, ** < 0.001, *** < 0.0001.

## 3. Results

### 3.1. Clinical Observations

**Figure 1:**
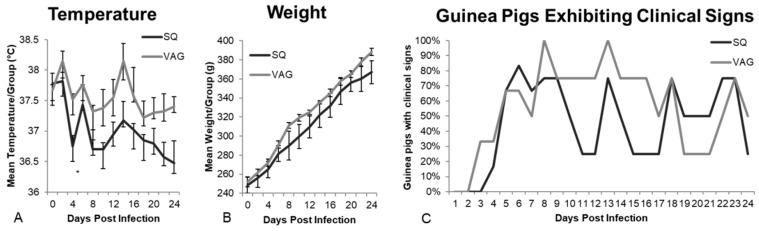

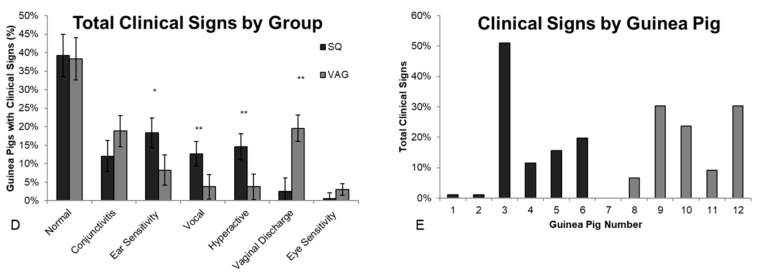
Clinical signs of infection following subcutaneous (SQ) and intravaginal (VAG) inoculation with 1 × 10^6^ PFU ZIKV MR766. (**A**) Temperature (average/group, * *p* < 0.01); (**B**) Weight (average/group); (**C**) Percentage of guinea pigs exhibiting clinical signs, including conjunctivitis, ear sensitivity, vocalization, hyperactivity, vaginal discharge, or eye sensitivity; cyclical peaks began 8 dpi and repeated every 5 days; (**D**) 291 total clinical observations were recorded over 24 days. The number of times each clinical sign was observed was recorded and is shown as percentage of guinea pigs in each group exhibiting specific clinical signs (* *p* < 0.05, ** *p* < 0.01); (**E**) Total clinical signs (excluding “normal” observations) recorded for each guinea pig.

The guinea pigs were observed for 24 days post infection (dpi) for clinical signs of ZIKV infection. Although none of the guinea pigs’ temperatures exceeded the normal range (37.2–39.5 °C), vaginally (VAG)-infected guinea pigs had higher temperatures than subcutaneously (SQ)-infected guinea pigs, which was statistically significant at 4 dpi (Figure 1A, *p* < 0.01). All guinea pigs gained weight at similar rates following infection (Figure 1B).

Based on clinical scoring, using a 5-point severity scale, ZIKV inoculation resulted in mild infections in both groups, although wide variability occurred among individual guinea pigs. Although mild, clinical signs were most apparent from 3–8 dpi and cyclical peaks of clinical signs occurred every five days thereafter (e.g., 8, 13, 18, 23 dpi, Figure 1C). While mild clinical signs of infection have been reported in previous guinea pig models, ours is the first to demonstrate a cyclical pattern to their nature. Over the 24-day observation period, 291 clinical observations were recorded; 39% of the 158 observations from the SQ group and 38% of the 133 observations from the VAG group were categorized as “no clinical signs” or “normal” (Figure 1D). Although we did not observe many classical overt signs of severe infection (e.g., ruffled fur, immobility, weight loss), some discrete signs of distress were observed in multiple animals. Conjunctivitis was observed in both SQ (12%) and VAG (19%) infected animals. However, guinea pigs infected SQ had a more frequent occurrence of ear sensitivity when tympanic temperatures were taken (*p = 0.014*), vocalization during handling (*p = 0.008*), and hyperactivity (*p = 0.002*) compared to the VAG infected group. In contrast, VAG infected guinea pigs had a significantly greater occurrence of vaginal discharge compared to SQ infected animals (*p* < 0.001 Figure 1D). Substantial variability was observed among all guinea pigs, regardless of inoculation route, with some animals showing minimal signs (Figure 1E). One guinea pig, GP#3, displayed the greatest number of observed signs of infection in the SQ group (GP#1-6), while GP#1 and GP#2 showed minimal signs of infection, which is consistent with ~20% of humans developing clinical disease from ZIKV infection. In the VAG group (GP#7-12), all but one guinea pig (GP#7) displayed signs of infection (Figure 1E). Determining the specific route of infection of humans in epidemic and endemic areas is rarely possible, thus the percentage of humans developing clinical symptoms following vaginal infection is not known. Therefore, the significance of these differences in relation to humans is challenging to interpret.

### 3.2. ZIKV Viral Shedding

**Figure 2:**
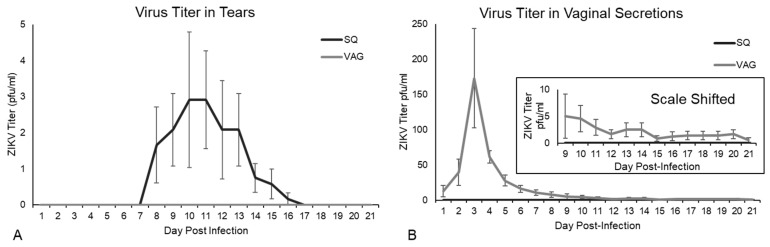
Viral shedding in guinea pigs inoculated subcutaneous (SQ) and intravaginal (VAG) with 1x10^6^ PFU ZIKV MR766. Virus titer, determined by plaque assay on Vero E6 cells, detected in: (**A**) tears; (**B**) vaginal secretions. Inset shows the graph with the Y-axis shifted to show average titer in vaginal secretions through 21 dpi in VAG infected animals.

Guinea pigs infected SQ shed infectious ZIKV in tears from 7-17 dpi, with peak shedding at 10 dpi (up to 10 PFU), although VAG-infected guinea pigs did not have detectable infectious ZIKV in tears (Figure 2A). Shedding of infectious ZIKV in tears after SQ injection (footpad) in immunocompromised or immunosuppressed mice has been demonstrated by Miner et al., who also noted the development of pan-uveitis [62]. Guinea pigs infected VAG shed infectious ZIKV in vaginal secretions throughout the study period, from 1–21 dpi, with peak shedding at 3 dpi (up to 425 PFU) (Figure 2B). The early peak of viral shedding in vaginal secretions at 3 dpi is consistent with local replication of the virus in vaginal or cervical tissues, prior to hematogenous spread to secondary tissues. Similar results have been documented in humans where infectious ZIKV has been recovered from vaginal secretions up to 10–14 days post symptom onset (pso) [28,63], although vaginal swabs have not been assessed the first few days following infection in humans to determine if very early viral shedding occurs. In contrast, no infectious virus was detected in vaginal secretions from guinea pigs inoculated SQ (Figure 2A). Overgrowth of the microflora of the oral cavity prevented successful plaque assay of oral swab samples.

### 3.3. ZIKV in Guinea Pig Tissues

**Figure 3:**
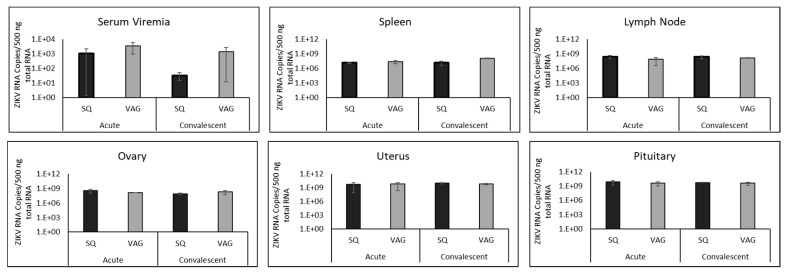
Viral load detected by RT-qPCR in serum and tissues of guinea pigs inoculated subcutaneously (SQ) or vaginally (VAG) with ZIKV at acute (7 dpi) and convalescent (37 dpi) time points.

A low-level serum viremia (1.09 × 10^3^–3.47 × 10^3^ ZIKV RNA copies/500 ng total RNA) was detected in both groups of guinea pigs during acute infection (Figure 3). This low-level viremia was still present in both groups of guinea pigs during convalescence (3.25 × 10^1^–1.36 × 10^3^ ZIKV RNA copies/500 ng total RNA), although reduced in SQ-infected guinea pigs. Wide variability was detected among all animals; thus, no statistically significant differences in viremia were found between the guinea pig groups during acute or convalescent time points. ZIKV RNA copy numbers in all tissues tested, other than the cerebellum of SQ-infected guinea pigs during the acute phase, were higher than serum viremia at both time points (7 dpi and 37 dpi) in both groups (Figure 3).

Similar ZIKV RNA loads were found in tissues of guinea pigs following SQ and VAG infection (Figure 3). Viral load detected during acute infection (7 dpi) did not change substantially by the convalescent time point (37 dpi), suggesting an extended period of time is required for clearance of viral RNA even though no signs of disease were present at the end point of the study. However, an increase in ZIKV RNA copies was detected in the spleens of VAG-infected guinea pigs from acute (2.41 × 10^7^) to convalescent (1.14 × 10^8^) time points, and a one-log higher copy number of ZIKV was detected in the spleens of VAG compared to the SQ group (*p = 0.0141*) at the convalescent time point. ZIKV RNA detected in lymph nodes was similar to that found in spleens (Figure 3), although no statistically significant differences were found.

The ovaries and uteri of both guinea pig groups contained ZIKV during both time points (Figure 3). Surprisingly, there was no statistically significant difference between the guinea pig groups at either time point due to inoculation route. Greater ZIKV copy numbers were noted in convalescent uteri (9.59 × 10^9^ SQ, 5.75 × 10^9^ VAG) than convalescent ovaries (7.65 × 10^7^ SQ, 1.87 × 10^8^ VAG) in both groups (p = 0.025 SQ; p = 0.021 VAG), suggesting a higher level of ZIKV persistent replication in the uterus during infection. The pituitary glands in each group supported robust ZIKV replication during acute infection (8.06 × 10^9^ SQ and 4.07 × 10^9^ VAG^,^), which continued into the convalescent period in both groups (5.37 × 10^9^ SQ and 3.76 × 10^9^ VAG), suggesting that ZIKV infection may affect glandular function.

**Figure 4:**
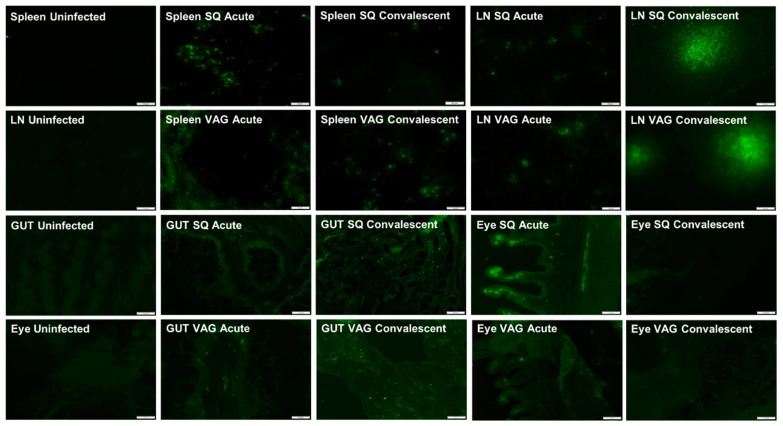
Representative immunofluorescence images of spleens, lymph nodes (LN), genitourinary tracts (GUT), and eyes from guinea pigs inoculated SQ or VAG with ZIKV at acute (7 dpi) and convalescent (37 dpi) time points. GUT images are from the uterus, which was the only part of the GUT we identified as positive for ZIKV antigen. Eye images show ciliary bodies, which were positive for ZIKV antigen.

ZIKV antigen was detected by immunofluorescence (IF) staining in the spleen and cervical lymph nodes at both acute and convalescent time points in SQ- and VAG-infected guinea pigs, with minimal visible differences between the groups (Figure 4). At the convalescent time point, ZIKV antigen was detected in diffuse vibrant clusters of cells, as opposed to distinct individual cells at the acute time point, suggesting possible cell-to-cell spread within lymph nodes (Figure 4).

The genitourinary tract (GUT) of both groups were IF stained for ZIKV to determine if ZIKV persisted in tissues through the convalescent time point. ZIKV antigen was detected in the GUT, specifically in the uterine wall, with virus persisting into the convalescent time point (Figure 4). At 7 dpi, ZIKV antigen was found localized to the uterus after VAG infection. ZIKV antigen was noted to be heaviest in VAG-infected guinea pigs at both time points compared to SQ-infected guinea pigs.

ZIKV antigen was observed in the eye of both SQ and VAG infected animals at both time points, but most prominently in the ciliary body of SQ infected guinea pigs during acute infection (Figure 4). Although present in VAG-infected animals, ZIKV antigen was noted to be heaviest in SQ infected guinea pigs compared to VAG-infected guinea pigs. This comports with our finding that guinea pigs infected SQ shed infectious ZIKV in ocular secretions. Similar immunofluorescent localization of ZIKV in the ciliary body has been reported in immunocompromised mouse models [64]. Although we did not detect substantial histopathology consistent with inflammation in the eyes of our adult guinea pigs, histopathological signs of inflammation have been localized to the ciliary bodies of fetal rhesus macaques (vertical transmission) and in a case series assessing congenital ZIKV syndrome in humans [65]. It is worth noting that no infectious virus was recovered from ocular secretions at acute or convalescent time points in VAG-infected guinea pigs, even though ZIKV viral RNA was detected in the eyes of this group, although less than after SQ infection. This suggests the possibility that a more robust viral replication is occurring in tissues that provide ocular secretions, such as the lacrimal glands.

### 3.4. ZIKV in Guinea Pig Nervous Systems

**Figure 5:**
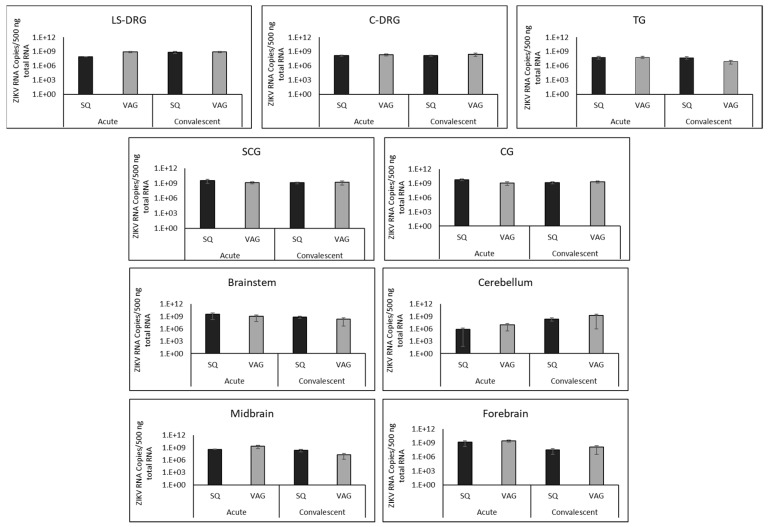
Viral load detected by RT-qPCR in nervous system of guinea pigs inoculated subcutaneously (SQ) or vaginally (VAG) with ZIKV at acute (7 dpi) and convalescent (37 dpi) time points. LS-DRG (lumbosacral dorsal root ganglia, sensory), C-DRG (cervical dorsal root ganglia, sensory), TG (trigeminal ganglia, sensory), SCG (superior cervical ganglia, sympathetic), CG (ciliary ganglia, parasympathetic).

Sensory ganglia (LS-DRG, C-DRG, and TGs) of the peripheral nervous system of both groups supported stable and persistent ZIKV RNA at both time points, with no significant differences between routes of infection. A slightly lower ZIKV copy number was detected in TGs from VAG- infected guinea pigs at the convalescent time point, although the results did not reach statistical significance (Figure 5).

Autonomic ganglia (SCG and CG) also contained similar ZIKV copy numbers following SQ and VAG infection, detectable during both acute and convalescent time points (Figure 3). These ganglia, in both groups, at both time points, supported ZIKV copy numbers slightly higher than those detected in sensory ganglia (1.24 × 10^9^–5.59 × 10^9^ RNA copies/500ng total RNA). There was no significant difference between ZIKV copy numbers in sympathetic (SCG) versus parasympathetic (CG) ganglia.

Structures throughout the brains of both SQ and VAG infected guinea pigs had high levels of detectable ZIKV RNA at acute and convalescent time points (Figure 5). ZIKV RNA copies decreased approximately 10-fold from acute to convalescent time points in brainstem (1.1–3.3 × 10^9^ acute to 2.1-7.5 × 10^8^ convalescent), midbrain (3.9 × 10^8^–2.0 × 10^9^ acute to 2.0 × 10^7^–2.2 × 10^8^ convalescent), and forebrain (1.6–2.6 × 10^9^ acute to 23.2 × 10^7^–1.3 × 10^8^ convalescent). In contrast, the cerebellum had fewer ZIKV RNA copies during acute infection (7.2 × 10^5^ SQ and 8.6 × 10^6^ VAG) compared to the convalescent time point (2.4 × 10^8^ SQ and 1.7 × 10^9^ VAG). In fact, the cerebellum contained lower copy numbers of ZIKV RNA than any other region of the nervous system during acute infection.

**Figure 6:**
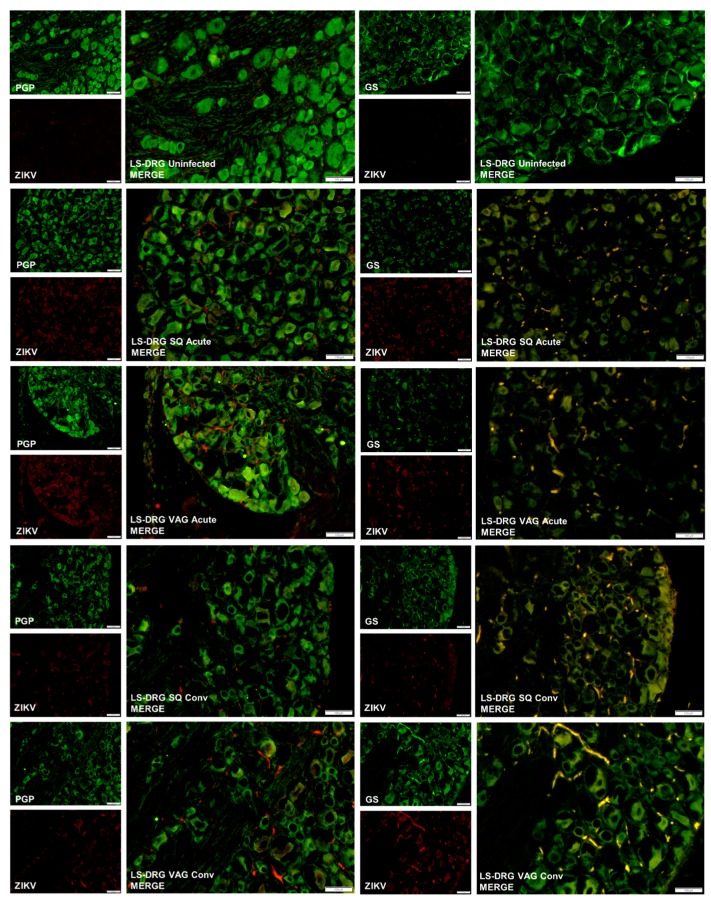
Representative immunofluorescence images of lumbosacral dorsal root ganglia (LS-DRG) from uninfected guinea pigs, and guinea pigs inoculated subcutaneously (SQ) or vaginally (VAG) with ZIKV at acute (7 dpi) and convalescent (37 dpi) time points. Neurons were immunostained for the sensory neuronal marker PGP9.5 (green) or the satellite glial cell marker glutamine synthetase (GS, green), and ZIKV (red). Merged images are shown enlarged to show co-localization of ZIKV with satellite glial cells, not sensory neurons. Note the morphological changes of satellite glial cells between uninfected (upper right) and ZIKV infected guinea pigs.

We previously demonstrated that cultured primary adult mouse sensory DRGs became infected with ZIKV and persistently released infectious virus for at least five days without dying [32]. We also determined that satellite glial cells (SGCs) in those cultures became infected and were killed by the virus within 24 h post inoculation. Thus, we hypothesized that the LS-DRG may be an alternative reservoir of persistent virus that could be shed in genital secretions, since the LS-DRG innervates the GUT. Thus, we assessed the presence of ZIKV in the LS-DRG to determine if neurons or SGCs became persistently infected with ZIKV following SQ or VAG infection. We detected ZIKV antigen by IF in the LS-DRG in both groups (Figure 6). During acute infection (7 dpi), ZIKV was detected by immunofluorescence surrounding sensory neurons within the ganglia and co-localizing with satellite glial cell (SGC) marker glutamine synthetase (GS). However, ZIKV was not detected within the neurons themselves, which were visualized by immunofluorescence for sensory neuronal marker PGP9.5. ZIKV persisted within the ganglia through the convalescent time point, particularly in the VAG-infected animals. SGCs normally wrap around sensory neurons within the ganglion (see LS-DRG Uninfected GS in Figure 6) but in ZIKV infected animals, regardless of route of inoculation or time point, the morphology of SGCs was substantially altered compared to uninfected animals, suggesting viral destruction of the SGCs. In contrast to the findings of our in vitro study, the sensory neurons were not infected in LS-DRG. ZIKV antigen was also detected in the SGCs of the C-DRG following SQ infection, but not in neurons. ZIKV antigen was found in C-DRG in only one of the VAG-infected guinea pigs during the acute time point.

**Figure 7:**
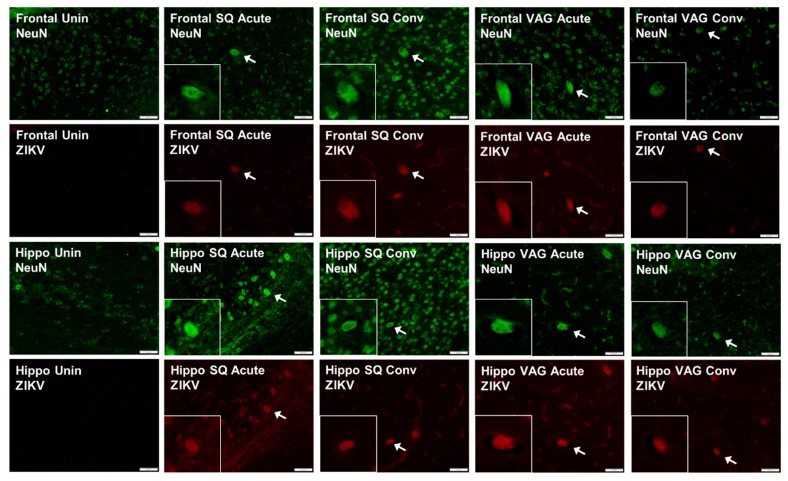
Representative immunofluorescence images of brain, including cortex in the frontal lobe (Frontal) and hippocampus (Hippo) from uninfected guinea pigs and guinea pigs inoculated subcutaneously (SQ) or vaginally (VAG) with ZIKV at acute (7 dpi) and convalescent (37 dpi) time points. Neurons were immunostained for the neuronal marker NeuN (green) and ZIKV (red). Insets are 200% enlargements of infected neurons, depicted by the white arrow, showing altered morphology.

Within the central nervous system, ZIKV antigen was observed in the brains of both groups at both acute and convalescent time points. Infection was diffuse throughout the brains of both groups, with fluorescence most notably in cortical tissues in the frontal lobe and in the hippocampus (Figure 7). Within each of these regions of the brain, isolated neurons identified as positive for ZIKV showed an altered morphology, substantially larger than nearby uninfected neurons and surrounded by a “halo”. This localization of ZIKV has been observed in a rhesus macaque model of infection (pregnant mother and fetus), as well as in human fetuses [17,66]. Histopathology, suggesting ZIKV localization to cortical tissues, has also been demonstrated in numerous murine models [36,38,62].

## 4. Discussion

Numerous murine models exist for the study of ZIKV infection. However most of these models use immunodeficient or immunosuppressed mice, many lacking an intact IFN pathway, which may influence the pathogenesis of infection (e.g., severe viremia, disease, frequent death) in a manner not applicable to that found in immunocompetent humans (e.g., asymptomatic infection, self-limiting illness, rare death) [40,67,68,69,70]. Several immunocompetent non-human primate models have been used to study the pathogenesis of ZIKV infection, however these models are limited due to their prohibitive cost, resulting small sample sizes, reduced statistical power, and requirements for specialized facilities and staff [44,45,46,47,48]. Due to concerns of sexual and vertical transmission and neurological sequelae of ZIKV infection, an immunocompetent small animal model is warranted.

A guinea pig model of ZIKV infection presents an attractive alternative to the above models due to the physiologic similarities between humans and guinea pigs, which include reproductive physiology and estrous cycle, and homology between immune systems (major histocompatibility molecules (MHC), complement systems, IFNγ pathways, IL-8/12 receptors, and CD8 sequences) [51]. Other attractive characteristics of the guinea pig include the ability to establish infection in an immunocompetent host, general ease of handling and maintenance, utilization of larger sample sizes, and availability of immunological assays and techniques [51]. Additionally, guinea pigs have been used as reliable models of flavivirus infection (e.g., Japanese encephalitis virus), as well as for sexual transmission studies (herpes simplex virus) and congenital syndrome caused by vertical transmission (cytomegalovirus) [54,55,71,72,73,74]. In this study, we demonstrate successful infection and persistence of ZIKV in immunocompetent female Hartley guinea pigs after a physiologically relevant inoculation (1 × 10^6^ PFU) in clinically significant routes of transmission (SQ, VAG), which mimic mosquito-borne and sexual transmission.

ZIKV infection was established in all guinea pigs regardless of route of infection. Minimal clinical signs of infection were observed, although subtle signs were noted, such as ear sensitivity, vocalization, and hyperactivity. This is consistent with human infection, as the majority (~80%) of ZIKV infections in humans are asymptomatic [75,76,77,78]. The classic maculopapular rash observed in some humans with symptomatic ZIKV infection was not observed in our guinea pigs, and has not been reported in other guinea pig models. Dermatological manifestations have only been reported in non-human primates around injection sites and more recently in tree shrews, although the tree shrews did not demonstrate any other signs of ZIKV infection such as fever or weight loss [45]. The mechanism for the development of skin rash associated with ZIKV infection is not fully understood. More severe/overt signs of disease have been elicited in guinea pigs by inoculation with more contemporary ZIKV strains (e.g., PRVABC59) [56], suggesting that these animals may be useful for investigating pathogenicity differences between ZIKV strains. We are the first to observe a five-day cyclical/undulating nature of clinical signs of infection, although the clinical significance of this observation relative to humans is not clear. Also, our studies are the first to compare SQ and VAG routes of infection, including the observations of the increase in vaginal discharge in animals inoculated vaginally, which may be a factor in potential sexual transmission of ZIKV.

A low-level viremia was detected via RT-qPCR in the serum of both groups throughout the study. Most current models were unable to detect viremia beyond 5 dpi; however, one model reported detection of low-level viremia up to 14 dpi, and one reported no detection at any time point [56,57,58,59]. Our detection of a sustained and persistent low-level serum viremia in both SQ and VAG infected groups up to 37 dpi represents the longest detection of serum viremia in a guinea pig model. This finding is consistent with detection of ZIKV viremia in whole blood samples in humans from 14 to 100 days, for a median duration of 22 days, while another serosurvey showed viremia for up to 8 weeks in some patients [79,80]. Localization of ZIKV replication to secondary lymphoid organs (spleen, lymph nodes), genitourinary tract (uterus, ovary), brain (brainstem, cerebellum, midbrain, forebrain, pituitary gland), and eyes agree with results reported in murine models, non-human primate models, and human case reports [35,37,40,44,56,57,65,81]. However, the only statistically significant difference we identified in tissue tropism between SQ and VAG routes of infection, based on tissue viral loads, was in spleens at convalescence (VAG > SQ), suggesting that ZIKV may be cleared more quickly after mosquito-borne transmission than after sexual transmission. This is also supported by the higher levels of ZIKV RNA we detected in serum at the convalescent time point in VAG infected compared to SQ infected animals. It is interesting to note the lower ZIKV RNA copy number in the cerebellums in both groups compared to any other region of the brain during acute infection, indicating a delay in entry or replication in the cerebellum for an unknown reason.

Infectious ZIKV was recovered from vaginal secretions from 1-21 dpi and from tears 7-17 dpi, with peak recovery at 3 dpi and 10 dpi, respectively. Our recovery of ZIKV from secretions is consistent with the recovery of ZIKV from vaginal secretions in humans from 10-14 days pso and in tears up to 30 days pso [28,63,82]. Interestingly, ZIKV was recovered from vaginal secretions in only VAG-infected guinea pigs and from tears in only SQ-infected guinea pigs, even though we detected ZIKV antigens in similar tissues in both groups and found no statistically significant differences in ZIKV RNA copy number in ocular or genitourinary tissues between the groups. Additionally, vaginal secretions supported a longer period of viral shedding and higher viral titers during peak shedding than tears, which may indicate a more robust and prolonged viral replication locally in VAG-infected animals. The increased vaginal discharge we noted in VAG-infected guinea pigs may contribute to higher shedding rates of infectious ZIKV in this group compared to SQ-infected animals, which may increase the risk of sexual transmission.

With respect to ZIKV shedding in tears, Deng et al. also isolated infectious ZIKV from the tears of SQ- and intranasally-infected guinea pigs, demonstrating contact transmission of ZIKV between SQ-infected guinea pigs and their naïve cage mates, potentially mediated through viral shedding in tears [57]. Our recovery of infectious ZIKV in tears from 7-17 dpi (albeit at low titers), combined with the detection of ZIKV RNA in tears of both index and contact animals by Deng et al., provides additional evidence to suggest contact transmission can occur in animal models by viral shedding in tears. As an extension of these findings, it is worth noting a case report in which contact transmission of ZIKV is suspected to have occurred between an elderly patient with a fatal ZIKV infection with high serum viremia and an otherwise healthy family member participating in his care who came into contact with the patient’s tears while not wearing personal protective equipment, subsequently developing a maculopapular facial rash and ZIKV antigenuria [83]. Taken together, these results suggest contact transmission of ZIKV via tears can occur between humans in certain rare instances.

We set out to determine if there were differences in tissue tropism and sites of ZIKV persistence that could contribute to extended periods of viral shedding in genital secretions that could contribute to sexual transmission, as well as vertical transmission. We had previously speculated that ZIKV persistence in LS-DRG, which innervate the GUT, may provide an alternative reservoir for viral shedding in genital secretions, particularly after sexual transmission [32]. We did not find significant differences in viral load or viral antigen in DRG following SQ or VAG infection, nor did we find significant differences in the GUT. However, we determined that ZIKV persists in the DRG and uterus following both SQ and VAG infection. To our knowledge this is the first in vivo animal model to investigate the role of sensory and autonomic ganglia in the maintenance of ZIKV infection. ZIKV antigen was localized to satellite glial cells (SGCs) surrounding the sensory neurons within LS-DRG in both groups during acute infection by 7 dpi, and the ganglia remained infected for at least 37 dpi. SGCs wrap around sensory neurons within the ganglia, providing support for the neurons as well as protection, forming a barrier between capillary endothelial cells and neurons within the ganglia and thus preventing access of blood-borne pathogens to the neurons. Based on our previous in vitro studies, in which SGCs were destroyed and naked neurons became persistently infected with ZIKV, we had anticipated that ZIKV would lytically infect SGCs, gaining access to sensory neurons within the DRG. DRGs from infected guinea pigs showed altered morphology and loss of SGCs surrounding sensory neurons within the ganglia, consistent with the destruction of SGCs we observed previously in primary DRG cultures [32]. However, the neurons themselves were not infected in either group. Detection of ZIKV antigen in SGCs, but not neurons of LS-DRG (or C-DRGs), suggests that ZIKV gains access to sensory ganglia through hematogenous dissemination but SGCs effectively prevent the virus from reaching and infecting the sensory neurons in vivo. Infection and destruction of SGCs surrounding neurons could disrupt synaptic transmission and potentially contribute to peripheral neuropathies. SGCs have been implicated in protection of neurons from blood-borne pathogens, as well as exacerbation of infection by mediating a robust inflammatory response within the ganglion. Although additional studies are needed to assess SGC survival, our results support a pathogenic model in which SGCs protect the sensory neurons from viremic ZIKV infection but contribute to viral spread and persistence in non-neuronal cells within the DRG. Additionally, our studies demonstrated ZIKV persistence in both sympathetic and parasympathetic autonomic ganglia. As autonomic ganglia innervate secretory glands and regulate release of secretions, persistence within autonomic ganglia also represents a previously undefined reservoir of persistent ZIKV that may contribute to viral shedding in secretions.

In summary, we sought to determine if route of infection influences pathogenesis of disease, tissue tropism and persistent reservoirs of ZIKV that may contribute to viral shedding. Although we did not identify differences in viral load or tissue tropism, route of infection contributed to substantial differences in viral shedding in secretions. Our studies support a pathogenic model in which ZIKV replicates locally at the site of infection, and then spreads hematogenously throughout the host. Following subcutaneous infection, simulating mosquito-borne transmission, ZIKV is more effective at shedding from ocular sections, although the site of persistence and mechanism are not completely clear. Following vaginal infection, local replication in the genitourinary tract induces increased vaginal secretions, which carry infectious virus that could contribute to sexual transmission. Since the majority of the ZIKV antigen that we found in the GUT after vaginal infection was localized to the uterine walls, the possibility exists that ZIKV sexual transmission may increase risk for the developing fetus during pregnancy. Further studies are needed to address pathogenic mechanisms of ZIKV by different routes of infection and the guinea pig model is well-suited for these endeavors.
